# The response of muscle progenitor cells to cutaneous thermal injury

**DOI:** 10.1186/s13287-017-0686-z

**Published:** 2017-10-17

**Authors:** Yusef Yousuf, Marc G. Jeschke, Ahmed Shah, Ali-Reza Sadri, Andrea-kaye Datu, Pantea Samei, Saeid Amini-Nik

**Affiliations:** 1 Institute of Medicine Science, University of Toronto, Toronto, Canada; 2 Sunnybrook Research Institute, Sunnybrook’s Trauma, Emergency & Critical Care (TECC) Program, Ross Tilley Burn Centre, M7-161, Lab: M7-140, 2075 Bayview Ave., Toronto, ON M4N 3 M5 Canada; 3 Laboratory in Medicine and Pathobiology, University of Toronto, Toronto, Canada; 4 Department of Surgery, Division of Plastic Surgery, University of Toronto, Toronto, Canada; 5 Ross Tilley Burn Centre, Sunnybrook Health Sciences Centre, Toronto, Canada

**Keywords:** Skeletal muscle, Muscle wasting, Thermal injury, Burn, Satellite cells, Pax7, Neutrophil, Muscle hypertrophy

## Abstract

**Background:**

Severe burn results in a systemic response that leads to significant muscle wasting. It is believed that this rapid loss in muscle mass occurs due to increased protein degradation combined with reduced protein synthesis. Alterations in the microenvironment of muscle progenitor cells may partially account for this pathology. The aim of this study was to ascertain the response of muscle progenitor cells following thermal injury in mice and to enlighten the cellular cascades that contribute to the muscle wasting.

**Methods:**

C57BL/6 mice received a 20% total body surface area (TBSA) thermal injury. Gastrocnemius muscle was harvested at days 2, 7, and 14 following injury for protein and histological analysis.

**Results:**

We observed a decrease in myofiber cross-sectional area at 2 days post-burn. This muscle atrophy was compensated for by an increase in myofiber cross-sectional area at 7 and 14 days post-burn. Myeloperoxidase (MPO)-positive cells (neutrophils) increased significantly at 2 days. Moreover, through Western blot analysis of two key mediators of the proteolytic pathway, we show there is an increase in Murf1 and NF-κB 2 days post-burn. MPO-positive cells were also positive for NF-κB, suggesting that neutrophils attain NF-κB activity in the muscle. Unlike inflammatory and proteolytic pathways, the number of Pax7-positive muscle progenitor cells decreased significantly 2 days post-burn. This was followed by a recovery in the number of Pax7-positive cells at 7 and 14 days, suggesting proliferation of muscle progenitors that accompanied regrowth.

**Conclusion:**

Our data show a biphasic response in the muscles of mice exposed to burn injury, with phenotypic characteristics of muscle atrophy at 2 days while compensation was observed later with a change in Pax7-positive muscle progenitor cells. Targeting muscle progenitors may be of therapeutic benefit in muscle wasting observed after burn injury.

**Electronic supplementary material:**

The online version of this article (doi:10.1186/s13287-017-0686-z) contains supplementary material, which is available to authorized users.

## Background

Severe burns result in a sustained inflammatory hypermetabolic response that typically lasts 9–12 months in humans [1, 2]. This leads to significant muscle wasting, weakness, and debilitation, which persists for the duration of the hypermetabolic response [1, 3]. In addition to burns, muscle wasting occurs in several diseases such as cancer-associated cachexia, cardiac failure, trauma, and chronic obstructive pulmonary disorder [4–8]. While there are several mechanisms put forward that attempt to explain muscle wasting following thermal injury, a clear picture of this pathology is yet to be defined [9].

During normal physiological conditions there is a balance between anabolic and catabolic pathways in muscle, and a key regulator of muscle homeostasis is skeletal progenitor satellite cells. For instance, satellite cells are not just implicated in myogenesis but also for muscle maintenance, remodeling, and regrowth [10, 11]. Pax7 is a transcription factor that regulates the proliferation of these satellite cells; hence, Pax7 is critical for maintaining muscle homeostasis [10, 12]. Post-burn changes in muscle protein synthesis are not as well defined as protein catabolism. Recent studies have shown that there is an increased number of Pax7-positive cells in muscle tissue following burn, indicating activation of a pro-regenerative response following cutaneous injury [13]. On the other hand, there are studies claiming decreased muscle protein synthesis and increased protein degradation after a burn injury [14, 15]. These studies obscure our insight into muscle homeostasis after a burn injury as there appear to be two competing processes occurring in muscle tissue: a tissue regenerative response and a hypercatabolic response. It is likely that the hypermetabolic response leads to dysregulation of protein degradation and synthesis that plays a role in muscle wasting observed post-burn.

Furthermore, the hypercatabolic response results in elevated serum levels of several inflammatory cytokines and growth factors for a prolonged period [2, 16–18]. One mechanism by which pro-inflammatory cells such as neutrophils exacerbate muscle atrophy after injury may be the nuclear translocation of NF-κB, a family of proteins that influence the expression of several proinflammatory genes [19]. Moreover, upon stimulation of neutrophils, NF-κB p65/RelA is recruited and translocated to the nucleus where it exerts its effects [19]. Activation of NF-κB in myoblasts leads to inhibition of the transcriptional expression of myogenic differentiation 1 (MyoD1) and myogenin, two genes that mediate myogenesis [20], which may exacerbate muscle wasting. Finally, inhibiting NF-κB prevents skeletal muscle atrophy in the soleus muscle of rats [21] and leads to the suppression of ubiquitin proteasome pathways involved in protein degradation [9]. Overall, neutrophils and NF-κB may be key inflammatory regulators of muscle atrophy after thermal injury.

Here we explore temporal changes in skeletal muscle after cutaneous burn injury in unrestrained mice and seek its mechanisms. We hypothesize that a local increase in inflammatory cells and cytokines such as neutrophils and NF-κB contribute to increased muscle atrophy during the acute phase (2 days) following thermal injury, and that a reduction in this inflammatory response will help mitigate the loss of muscle mass expected at later time points post-burn. By subjecting mice to a 20% total body surface area (TBSA) thermal injury, we seek to ascertain the mechanisms of muscle wasting post-injury.

## Methods

### Mice

All mice used were male, 8-week-old C57BL/6 mice. Twenty-four mice were randomly divided into four groups: sham, 2 days, 7 days, and 14 days post-thermal injury (*n* = 6 per group). The animal experiments were reviewed and approved by, and performed in accordance with, the guidelines and regulations set forth by the Sunnybrook Research Institute and Sunnybrook Health Sciences Animal Policy and Welfare Committee of the University of Toronto, Ontario, Canada. All procedures using animals were approved by the Sunnybrook animal care committee, approval #15-503(M-1) issued 20 November 2015 under the auspices of the Canadian Council on Animal Care.

### Burn injury

Animal procedures were reviewed and approved by the Sunnybrook Research Institute and Sunnybrook Health Sciences Centre at University of Toronto Animal Care and Use Committee. Mice were subjected to a full thickness scald burn. Briefly, animals were anesthetized with inhaled isoflurane and received an intraperitoneal buprenorphine injection (0.1 mg/kg). The dorsum of the animal was shaved and lactated Ringer’s solution was injected subcutaneously along the spine. The mice were placed on a mold that exposes the dorsum (20% TBSA) [22]. A 20% TBSA thermal injury was induced by exposing the dorsum of the animal to water preheated to 98 °C for 10 s. Following burn, the animals were placed in separate cages. The mortality rate of this scald burn is approximately 5%. Sham animals were anesthetized and received buprenorphine injection but did not receive a thermal injury.

### Muscle harvest and dry/wet muscle ratio

We dissected the gastrocnemius muscle from mice 2, 7, and 14 days after exposure to cutaneous thermal injury and from the sham mice for histological and protein analysis. The whole gastrocnemius muscle was weighed at the time of harvest to obtain the wet muscle weight. The dry muscle weight was obtained by dehydrating the whole gastrocnemius muscle for 5 days at 50 °C. The dry muscle weight was weighed. The dry muscle weight was divided by the wet muscle weight to obtain the dry/wet muscle ratio. For histology, muscle samples were tied to a support prior to excision to prevent contraction.

### Myofiber cross-sectional area analysis

For histological analysis, the gastrocnemius muscle was fixed in 10% formalin and sectioned in the transverse plane at 5 μm. Masson’s trichrome staining was performed using established protocols [12, 23]. Representative images of the gastrocnemius muscle sections were captured at 10× magnification. Blue color pixel count was performed using Adobe® Photoshop® software to quantify areas of intramuscular collagen and fibrosis. The cross-sectional area of individual myofibers was obtained through ImageJ® software. For each animal, the cross-sectional areas of multiple myofibers were counted in five images (field-of-views) and, subsequently, the average cross-sectional area was determined. We did not differentiate between type I and type II fibers when measuring muscle cross-sectional area.

### Western blot

Gastrocnemius muscle was harvested and protein was isolated from tissue lysates using RIPA lysis buffer. Protein concentrations were then measured using a bicinchoninic acid (BCA) assay as previously reported [24]. Briefly, 50 μg of each protein sample was separated by SDS-polyacrylamide gel electrophoresis, transferred to a nitrocellulose membrane, blocked with 5% milk in tris-buffered saline/0.1% Tween 20, and hybridized with the following primary antibodies: anti-Murf1 (1:1000, ThermoScientific), anti-NF-κB p65 (1:1000, Cell Signaling), anti-Pax7 (1:500, DHSB), anti-mTOR (1:1000, Cell Signaling), anti-MyoD 5.8A (1:1000, BD Biosciences), and GAPDH (1:5000, Cell Signaling). The membranes were then incubated with anti-rabbit or anti-mouse horseradish peroxidase (HRP)-conjugated secondary antibody (1:2500, Santa Cruz). Detection of the signal was accomplished using Western HRP chemiluminescence (ECL) reagents (Bio-Rad Laboratories) and imaging of the blots was performed using the ChemiDoc™ MP System (Bio-Rad). To analyze the blots, Image Lab™ Software (Bio-Rad) was used to quantify band intensity and calculate the absorbance ratio of the target protein compared to the loading control, GAPDH.

### Immunohistochemistry

Gastrocnemius muscle samples for histological analysis were collected and fixed in 10% formalin for 24 h and transferred to 70% ethanol. Samples were then embedded in paraffin and sectioned at 5 μm across the transverse plane. Paraffin-embedded slides were heated at 60 °C for 30 min, deparaffinized with citrosol, and rehydrated through a series of decreasing alcohol concentrations. Antigen decloaker solution (Biocare Medical) was preheated in an antigen decloaking chamber at 70 °C for 20 min before the slides were added. The slides were then heated at 100 °C in the antigen decloaking solution for 4 min, cooled to 60 °C, and washed with tap water. After blocking endogenous peroxidase activity with 3% H_2_O_2_ for 10 min, sections were incubated with the following primary antibodies: anti-Murf1 (1:800, ThermoScientific), anti-MPO (1:200, Abcam), anti-NF-κB p65 (1:200, Cell Signaling), and anti-Pax7 (1:100, DHSB). Slides were washed with washing buffer (0.05 M Tris–HCl, 0.15 M NaCl, and 0.05% Tween 20 in double distilled water). Sections were then incubated in MACH3 probe (Biocare Medical) for 15 min, washed, and MACH3 HRP polymer detection was added for 15 min. After washing again, betazoid diaminobenzidine (DAB) chromogen kits (Biocare Medical) were mixed and incubated for 10 min, or until a brown color was observed. Slides were rinsed in running tap water, stained with hematoxylin for 30 s, and washed and briefly differentiated in 1.5% acid alcohol. Slides were then placed in 0.1% sodium bicarbonate for 10 s and dehydrated in citrosol and alcohol solutions. Finally, slides were mounted and cover slipped with xylene-based aqueous mounting media (SHUR/Mount™).

For quantification, five different fields were randomly chosen for each sample. The sections were imaged via an optical microscope (Leica Microsystems) with 20× and 40× objective lenses. The percentage of positive cells for each target was determined by dividing the number of positive cells by the total number of nuclei in each histological field. The average ratio for each subject was considered. Negative controls without primary antibody but with DAB staining were prepared to confirm the staining observed.

### Immunofluorescence protocol

Frozen muscle samples were embedded in OCT and frozen in liquid nitrogen-cooled isopentane. The samples were cut perpendicularly via a cryostat (10-μm thickness). Sections were allowed to cool for 5 min at room temperature and subsequently fixed in 4% paraformaldehyde (PFA) for 5 min. Sections were washed with phosphate-buffered saline (PBS) and incubated with glycine solution to quench the PFA signal. After washing, sections were permeabilized in 0.25% Triton-X for 10 min and washed again. Sections were then incubated in blocking buffer (5% normal goat serum, 2% bovine serum albumin, and mouse-on-mouse blocking reagent-diluted PBS) for 1 h at room temperature. Sections were rinsed in PBS and incubated in primary antibody overnight at 4 °C. The following antibodies were used: laminin (1:200, rabbit, Abcam), anti-MPO (1:200, rabbit, Abcam), and anti-NF-κB p65 (1:200, mouse, Cell Signaling). Sections were rinsed with PBS and incubated in secondary antibody solution diluted in blocking buffer: goat anti-rabbit Alexa Fluor 488 (1:1000) and goat anti-mouse IgG1 Alexa Fluor 546 (1:1000). Sections were rinsed and mounted with fluorescent mounting media containing DAPI (Vector Laboratories). Samples were imaged with a Zeiss Apotome fluorescent microscope.

### Statistical analysis

Statistical analysis was performed using one-way analysis of variance (ANOVA). Data are represented as mean ± SEM (*n* = 6). *P* < 0.05 was considered as being statistically significant.

## Results

### Thermal injury induces a biphasic muscle response in mice

**Fig. 1:**
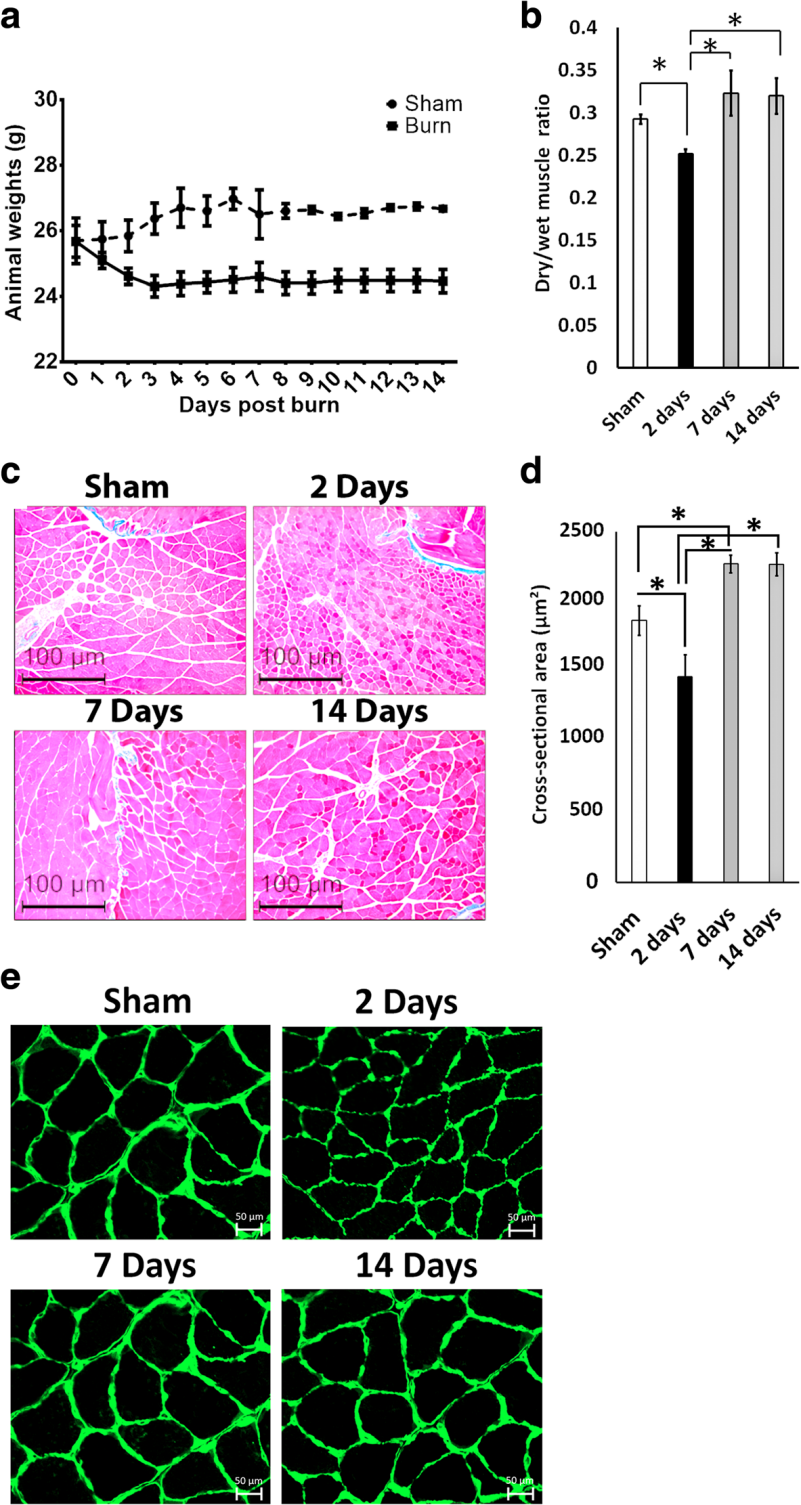
Thermal injury leads to a transient muscle atrophy and an increase in muscle mass. **a** Animal weights from day 0 to day 14 following thermal injury. **b** Muscle mass expressed as a dry/wet muscle ratio. **c** Representative images of Masson’s trichrome staining of mice gastrocnemius from the sham group, and 2 days, 7 days, and 14 days following cutaneous thermal injury. Images were obtained at 10× magnification and myofiber cross-sectional area (μm^2^) was measured for all time points. Both type I and type II fibers were included in the analysis. **d** Quantification of muscle cross-sectional area. **e** Representative laminin staining of myofibers via immunofluorescence. Images taken at 40× magnification. *P < 0.05

Animal weights in the burn groups decreased compared to the sham group, and this decrease was sustained until 14 days post-thermal injury (Fig. 1a). This loss of body weight can be attributed to increased lipolysis after thermal injury which significantly reduces fat mass [18, 25]. Previous studies have reported muscle atrophy in the gastrocnemius muscle 2 days after severe cutaneous thermal injury [26]. In concordance with these findings, we observed a decrease in the dry/wet muscle ratio 2 days after cutaneous burn injury compared to the sham group (Fig. 1b). We also observed a significant reduction in myofiber cross-sectional area at this time point (Fig. 1c and d), which is suggestive of muscle atrophy [27, 28]. Interestingly, a significant increase in myofiber cross-sectional area and dry/wet muscle ratio was observed at 7 days post-burn when compared to 2 days and the sham group (*P* < 0.05). (Fig. 1b and d). These changes in myofiber cross-sectional area can also be observed in Fig. 1e, where the myofiber borders are more clearly defined. This increase not only indicates muscle recovery, but also suggests transient compensatory muscle regrowth following a burn injury. Furthermore, we observed maintenance of this hypertrophic phenotype 14 days post-burn (Fig. 1c and d). We also investigated the potential for local intramuscular fibrosis following cutaneous burn [22, 29] using Masson’s trichrome staining of the gastrocnemius muscle, but we found no significant difference between the groups (data not shown).

### MURF1 is transiently upregulated in the muscle of mice subjected to thermal injury

**Fig. 2:**
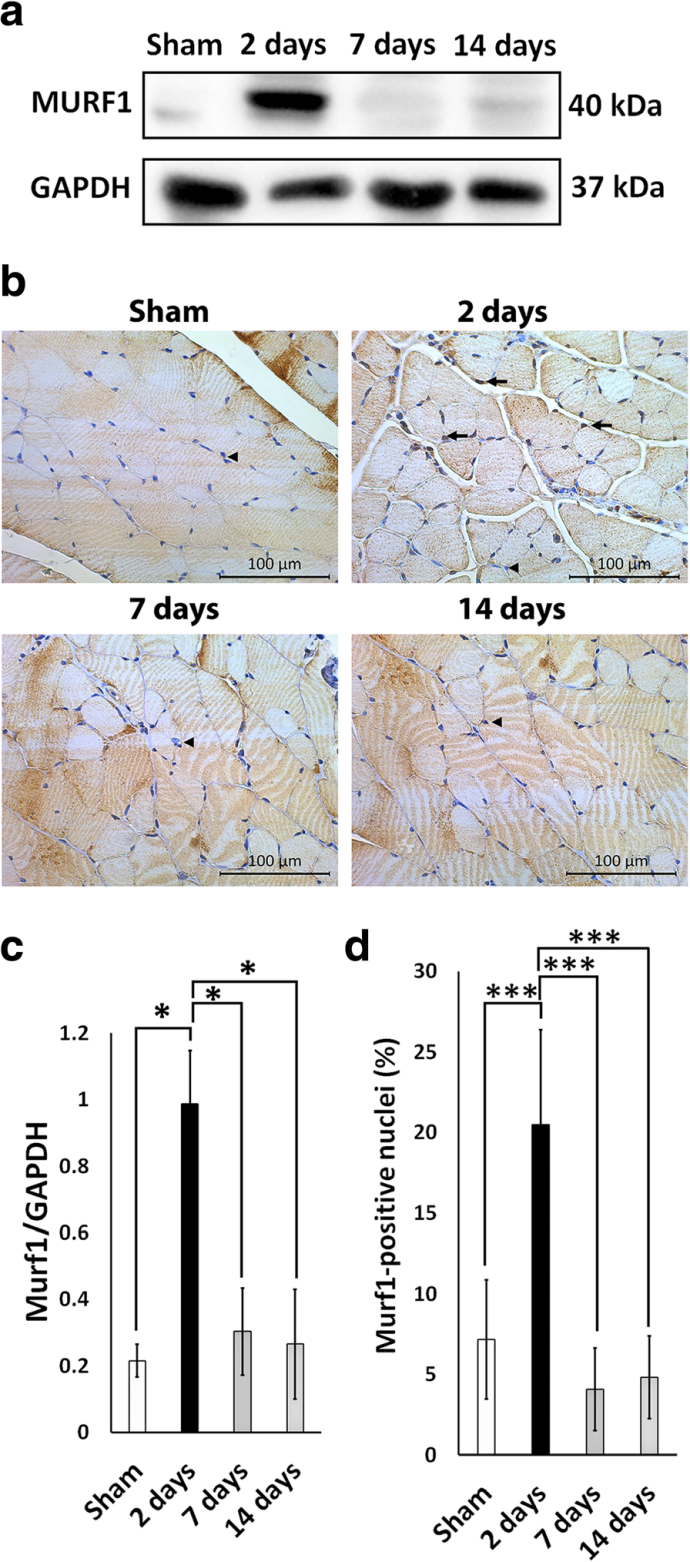
Temporal overexpression of Murf1 and accumulation of Murf1-positive nuclei in muscle 2 days post-burn. **a** Representative Western blot for Murf1. **b** Representative images of Murf1 immunohistochemistry. Images were obtained at 20× magnification. **c** Quantification of Murf1 protein expression. **d** Quantification of Murf1-positive nuclei in sham, 2 days, 7 days, and 14 days post-burn. *Arrows* indicate Murf1-positive nuclei and *arrowheads* indicate Murf1-negative nuclei. *P < 0.05, ***P < 0.001

*Murf1* expression is specifically increased in muscle atrophy associated with several physiological triggers such as limb immobilization and cancer cachexia [30–32]. The protein level of MURF-1 was significantly increased in muscle 2 days post-burn as quantified by calculating the ratio of MURF-1 to GAPDH at this time point (*P* < 0.05 vs sham, 7 days, and 14 days) (Fig. 2a and c). This overexpression coincided with significant muscle atrophy observed at 2 days post-burn. To further evaluate the spatial expression of MURF-1, we stained the muscle section with MURF1 antibody. There was an accumulation of MURF1-positive nuclei in muscle at 2 days post-burn signifying an increase in the transcriptional activity of Murf1 (*P* < 0.001 vs sham) (Fig. 2b and d). We could not determine a specific spatial pattern for MURF-1-positive cells as they were distributed randomly throughout the sections. MURF-1 has been reported to associate with the M- and Z-lines of the sarcomere in addition to its presence within myonuclei [33, 34]. We observed some MURF-1 staining within the fibers. Consistent with prior reports [26], these results suggest that muscle wasting post-burn is associated with an increased activity of Murf-1. Finally, both Western blot analysis and immunohistochemistry revealed no significant differences in Murf1 expression between sham, 7 days post-burn, and 14 days post-burn (Fig. 2a and b).

### Thermal injury results in a transient recruitment of neutrophils, but not macrophages, into muscle

**Fig. 3:**
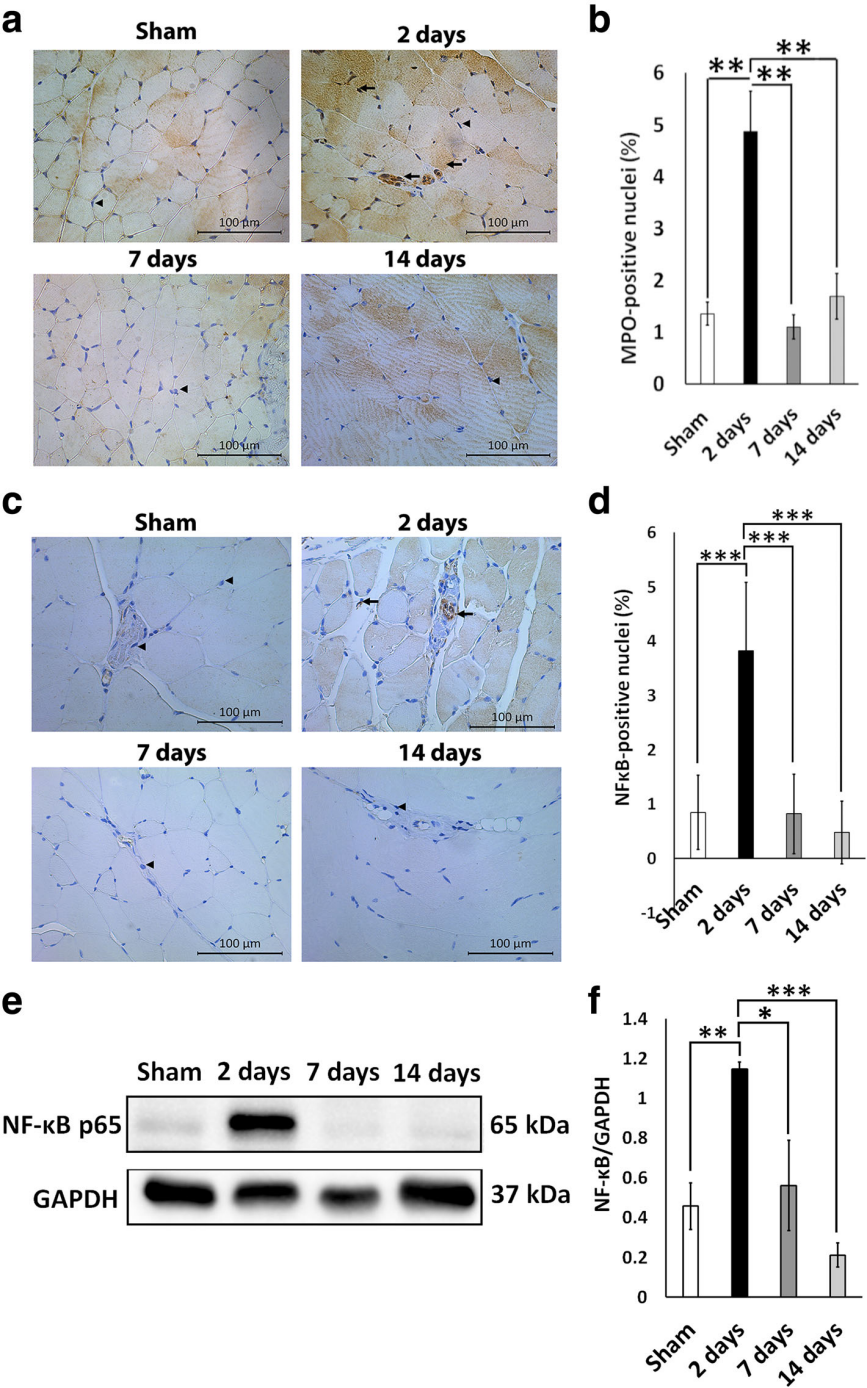
Thermal injury recruits neutrophils into the muscles and leads to a temporal overexpression of NF-κB and accumulation of NF-κB-positive nuclei in muscle 2 days post-burn. **a** Representative images of myeloperoxidase (*MPO*) immunohistochemistry. Images were obtained at 20× magnification. *Arrows* indicate MPO-positive nuclei and *arrowheads* indicate MPO-negative nuclei. **b** Quantification of MPO-positive nuclei in sham, 2 days, 7 days, and 14 days post-burn. **c** Representative images of NF-κB immunohistochemistry. Images were obtained at 20× magnification. *Arrows* indicate NF-κB-positive nuclei and *arrowheads* indicate NF-κB-negative nuclei. **d** Quantification of NF-κB-positive nuclei in sham, 2 days, 7 days, and 14 days post-burn. **e** Representative Western blot for NF-κB. **f** Quantification of NF-κB protein expression. *P < 0.05, **P < 0.01, ***P < 0.001

To investigate the local inflammatory effect of severe cutaneous burn on muscle, we looked for the presence of inflammatory cells in the gastrocnemius muscle. During the time course of the experiment we did not observe the presence of macrophages via immunohistochemistry for F4/80, a marker for mouse macrophages (Additional file 1). However, we observed a significant increase in the prevalence of myeloperoxidase (MPO)-positive nuclei 2 days post-burn (*P* < 0.01 vs sham) (Fig. 3a and b). MPO-positive nuclei were only present transiently, and their expression returned to control levels by 7 days post-burn (Fig. 3a and b).

### Cells which are positive for the active form of NF-κB are enriched in the muscle of mice 2 days following thermal injury

To ascertain the role of NF-κB in muscle wasting following thermal injury, we examined NF-κB activity post-burn via immunohistochemistry and Western blotting. There was an accumulation of NF-κB-positive nuclei in muscle at 2 days post-burn signifying an increase in the transcriptional activity of NF-κB (*P* < 0.01 vs sham) (Fig. 3c and d). Most of these NF-κB-positive nuclei were present within and around blood vessels, suggesting that acute inflammation led to activation and/or recruitment of cells positive for the active form of NF-κB. Western blot analysis and immunohistochemistry revealed no significant differences in NF-κB expression between sham, 7 days post-burn, and 14 days post-burn (Fig. 3e and f). However, protein expression of NF-κB was significantly increased in muscle 2 days post-burn as indicated by the absorbance ratio of NF-κB to GAPDH at this time point (*P* < 0.05 vs sham, 7 days, 14 days) (Fig. 3e and f). This overexpression coincided with significant muscle atrophy observed at 2 days post-burn (Fig. 1). Overall, these results show an association between NF-κB RelA/p65 and muscle atrophy during muscle wasting following thermal injury.

### MPO cells attain NF-κB activity at 2 days post-thermal injury

**Fig. 4:**
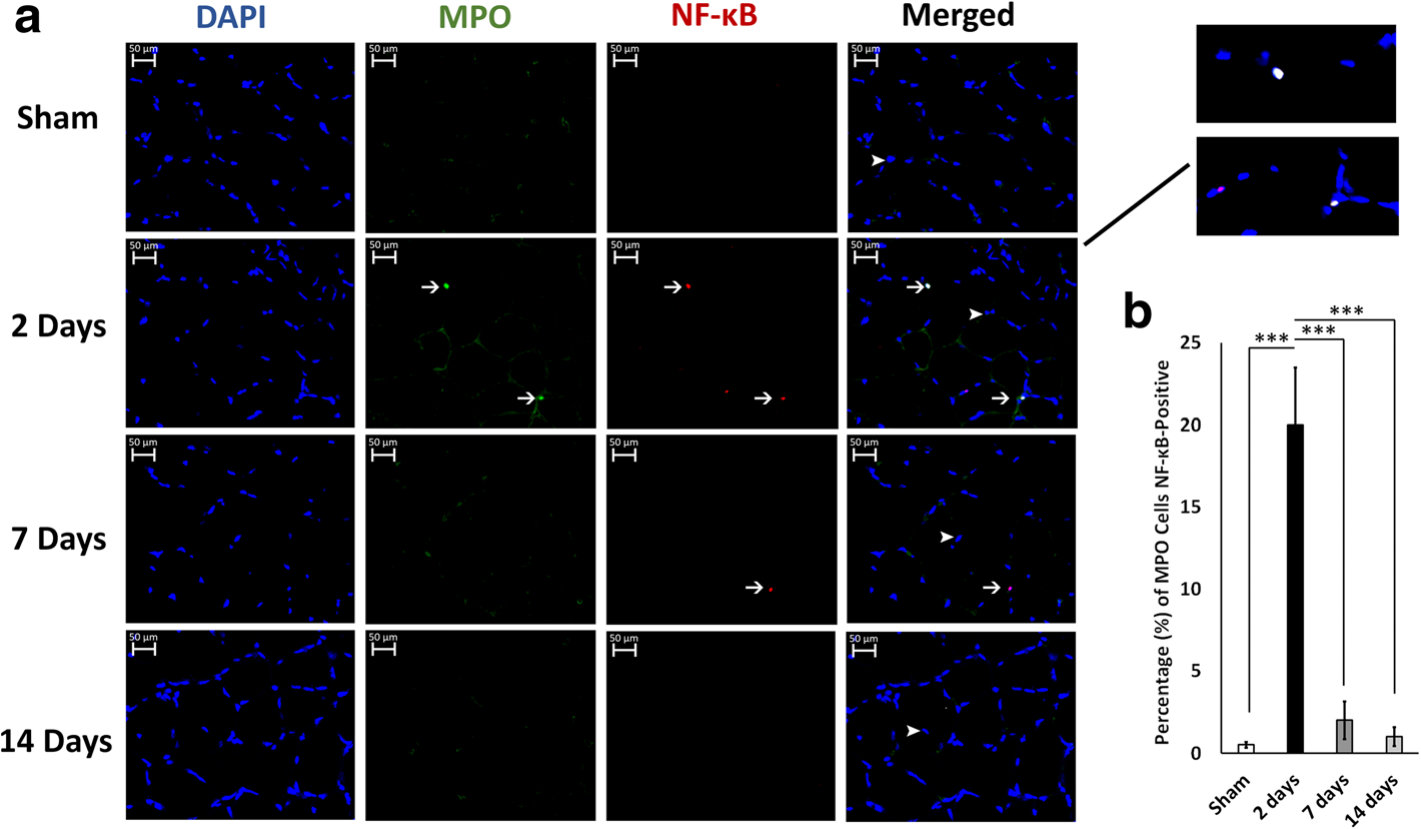
Neutrophils attain NF-κB activity in muscle at 2 days post-thermal injury. **a** Representative double immunofluorescence staining showing that myeloperoxidase (*MPO*) cells were also positive for NF-κB at 2 days post-thermal injury. *Arrows* indicate positive cells, and *arrowheads* show negative cells. Images taken at 40× magnification. **b** quantification of MPO cells that were also NF-κB positive. ***P < 0.001

Neutrophils can upregulate the expression of many genes, including those encoding cytokines. Increased activation of NF-κB in neutrophils has been associated with several inflammatory diseases such as acute lung injury, pulmonary disease, and sepsis [35–37]. Previous studies have shown that activating human neutrophils via the pro-inflammatory agonists lipopolysaccharide (LPS) and tumor necrosis factor (TNF)-α increases NF-κB activity in these cells [19, 38]. To examine whether neutrophils attain NF-κB activity during the inflammatory response we performed immunofluorescence for MPO and NF-κB RelA/p65. At 2 days post-thermal injury, we observed a significant increase in MPO and NF-κB double-positive cells (Fig. 4a). Approximately 20% of MPO cells showed co-localization with NF-κB (Fig. 4b). These results indicate that neutrophil infiltration into the muscle during the burn response is associated with NF-κB activity which may contribute to the muscle atrophy observed.

### Muscle progenitor cells are significantly downregulated 2 days post-thermal injury

**Fig. 5:**
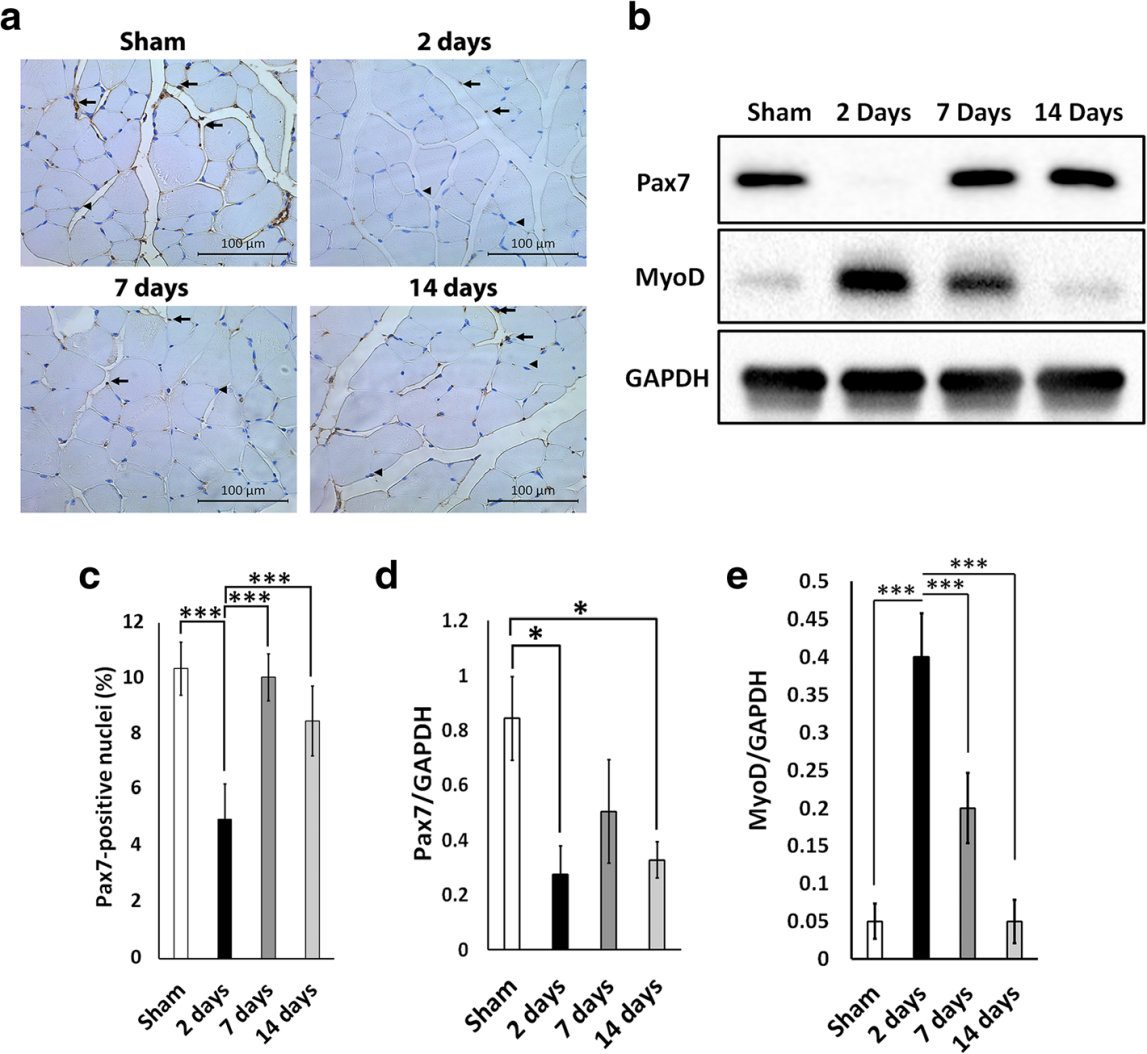
Muscle progenitor cells respond to thermal injury in a bi-phasic fashion. **a** Representative images of Pax7 immunohistochemistry. Images were obtained at 20× magnification. **b** Representative Western blot for Pax7 and MyoD. **c** Quantification of Pax7-positive nuclei in sham, 2 days, 7 days, and 14 days post-burn. *Arrows* indicate Pax7-positive nuclei and *arrowheads* indicate Pax7-negative nuclei. **d** Quantification for Pax7 Western blot. **e** Quantification for MyoD Western blot. *P < 0.05, ***P < 0.001

Protein expression of Pax7 was significantly reduced in muscle 2 days post-burn and 14 days post-burn (*P* < 0.05 vs sham) (Fig. 5b and d). There was no significant difference in Pax7 protein level between sham and 7 days post-burn (Fig. 5b). There was a significant decrease in the number of Pax7-positive nuclei at 2 days post-burn versus the sham group (*P* < 0.001 vs sham) followed by a recovery in the Pax7-positive pool at 7 and 14 days post-burn (*P* < 0.001 vs 2 days) (Fig. 5a and c). These data suggest that the number of muscle satellite stem cells dramatically decreases during the acute atrophic response to thermal injury. A transcription factor that regulates myogenic differentiation is MyoD. To examine changes in muscle differentiation we examined the MyoD protein levels. We found a significant increase in MyoD protein levels at 2 days post-thermal injury (Fig. 5b and e). It is likely that the decrease in Pax7 protein level and the increase in MyoD protein level observed at 2 days post-thermal injury is a result of muscle progenitors committing to myogenesis. The differentiation of muscle progenitors into new myofibers may have contributed to the recovery of muscle size observed at 7 and 14 days post-thermal injury.

### High frequency of NF-κB- and MURF1-positive nuclei in muscle tissue is associated with muscle atrophy

**Fig. 6:**
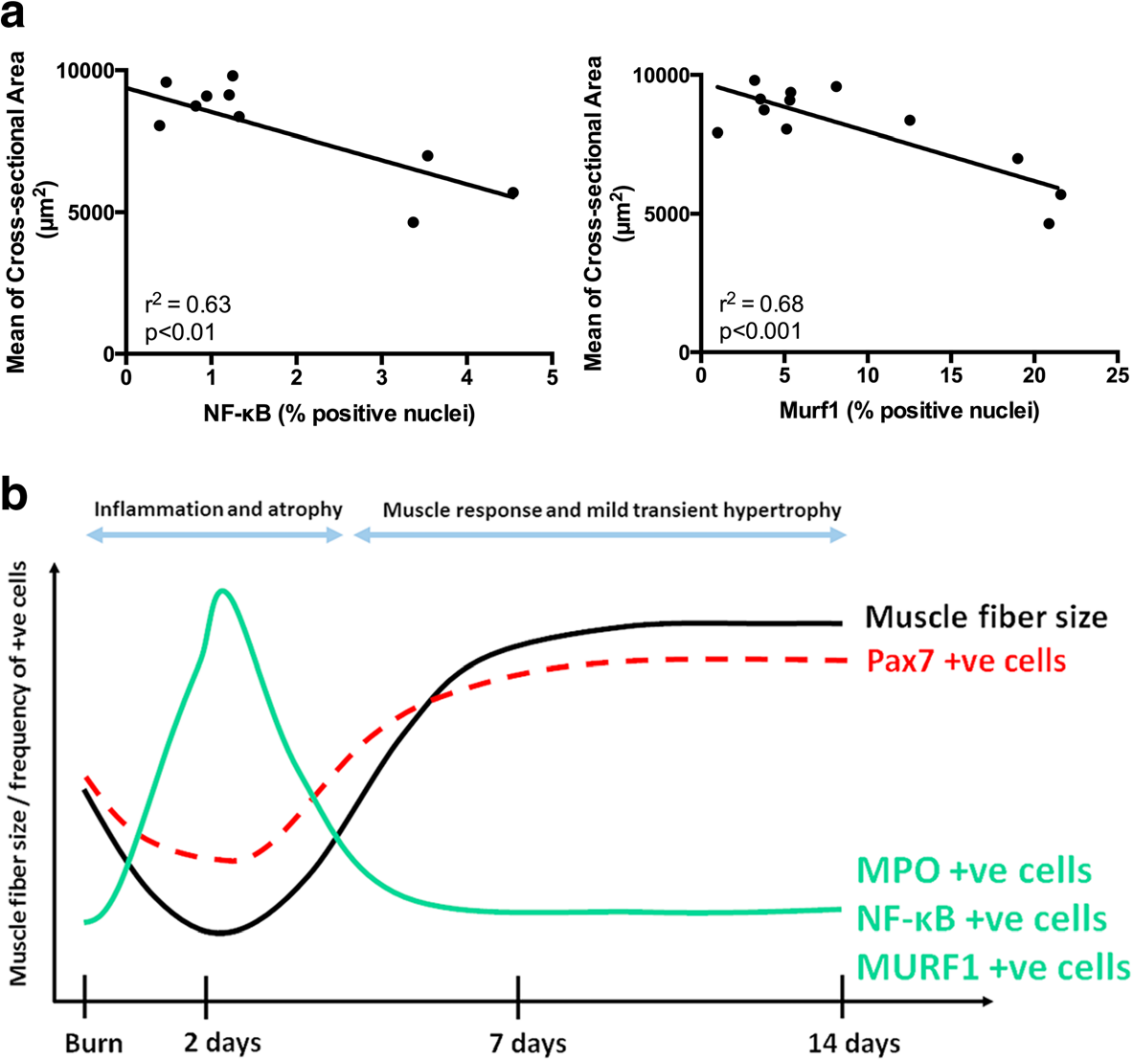
The abundance of NF-κB- and Murf1-positive nuclei in muscle tissue correlates with muscle atrophy. **a** Correlation plots between the percentage of NF-κB-positive nuclei and the mean of myofiber cross-sectional area (r^2^ = 0.63, *P* < 0.01), and the percentage Murf1-positive nuclei and the mean of myofiber cross-sectional area (r^2^ = 0.68, *P* < 0.001). Correlation analysis was performed using two-tailed Pearson correlation. The analysis includes mice from all treatment groups (sham and 2 days, 7 days, and 14 days post-burn). **b** Cellular and molecular cascades associated with muscle atrophy during the acute burn response and subsequent muscle regrowth. *MPO* myeloperoxidase

To confirm the association between muscle atrophy and NF-κB and Murf1 expression we performed a correlation analysis between the abundance of NF-κB- and Murf1-positive nuclei and the myofiber cross-sectional area. Animals from all treatment groups (sham, 2 days post-burn, 7 days post-burn, and 14 days post-burn) were included in the analysis. We report that the frequency of NF-κB-positive (*r* ^2^ = 0.63, *P* < 0.01) and MURF1-positive (r^2^ = 0.68, *P* < 0.001) nuclei in muscle tissue is inversely associated with myofiber cross-sectional area (Fig. 6a). This further suggests that NF-κB- and Murf1-positive cells contribute to muscle atrophy observed following thermal injury. A summary of the cellular and molecular cascades involved in muscle atrophy after a burn injury is summarized in Fig. 6b.

## Discussion

In this study, we initially observed muscle atrophy followed by compensatory regrowth in unrestrained mice after severe cutaneous thermal injury. Immediately post-burn, we observed an inflammatory response characterized by the presence of intramuscular MPO-positive cells and the pro-inflammatory marker NF-κB. Several studies have shown an accumulation of neutrophils in skeletal muscle after injury, which reaches peak concentration at 1 day and returns to control levels at 7 days post-injury [39–41]. These studies also indicate that neutrophils worsen muscle wasting. The correlation of MPO and NF-κB expression may be due to MPO-positive cells attaining NF-κB activity during the hypermetabolic response, which is associated with muscle atrophy. In our study, we show co-localization of MPO cells with NF-κB via immunofluorescence (Fig. 4a). It is likely that the recruitment of neutrophils results in nuclear translocation of NF-κB as shown in Figs. 3 and 4. This may lead to the secretion of cytokines that may change the microenvironment of skeletal muscle resulting in an activation of the muscle atrophy marker *Murf1* [26, 27] and a decrease in myofiber size. Further studies to investigate the causative relationship between MPO-positive cells and NF-κB expression are warranted based on their correlation shown by this study. NF-κB response elements are required for Murf1 gene activation during skeletal muscle atrophy in several diseases [9, 42], thus explaining why the protein expression of NF-κB and Murf1 mirror each other in our study (Fig. 5b).

Prior to this study, the role of NF-κB in muscle wasting post-burn was limited. A study found low levels of NF-κB in burn patients [43]. This is expected since NF-κB signaling in pro-inflammatory cells has been implicated in muscle atrophy and degeneration [44, 45], whereas downregulation of NF-κB is associated with enhanced resistance to atrophy and regeneration of muscle fibers [46]. The exact mechanisms by which NF-κB promotes muscle wasting remain ambiguous. In cancer-induced cachexia, NF-κB expression is associated with Pax7 dysregulation and muscle wasting [47]. A decreased prevalence of Pax7-positive satellite cells has been associated with myofiber dysfunction and muscle wasting muscle atrophy [48, 49], whereas their proliferation is associated with muscle regeneration and myofiber synthesis [50, 51]. Interestingly, our findings reveal that NF-κB expression coincides with the reduction in muscle fiber cross-sectional area and the downregulation of Pax7 expression at 2 days post-burn, an occurrence that is similar to that in cancer-induced cachexia. As the level of MPO and NF-κB decreases at 7 and 14 days post-burn, we observed a shift into a pro-regenerative muscle niche characterized by proliferation of Pax7-positive satellite stem cells and subsequent muscle restitution and hypertrophy.

Typically, in burn and cancer-induced cachexia there is a systemic effect that promotes muscle wasting for a prolonged period. In cancer-induced cachexia, NF-κB activation coincides with an increase in Pax7 expression [47]. In contrast, our study found that NF-κB coincides with a decrease in Pax7 expression, suggesting that the function of NF-κB differs between a burn injury and cancer-induced cachexia. In our burn study, we observed similar mechanisms of muscle atrophy; however, these mechanisms were only observed transiently and locally. Although Pax7 cells have been shown to be necessary for muscle hypertrophy [11], the relation between Pax7 activation and muscle protein synthesis is not clear since the activation and proliferation of satellite cells does not necessarily lead to increased protein synthesis in skeletal muscle.

Reduced satellite cell abundance was recently observed in pediatric burn patients; as such, our rodent data supports clinical findings [52]. The reduction of satellite cells following burn injury most likely effects the recovery of lean muscle mass and likely contributes to the muscle wasting that is characteristic of burn patients. In contrast to our results, a recent paper has shown increased Pax7 mRNA expression at 1, 3, and 7 days post-burn in mice [53]. The difference in the induction of Pax7 expression between the studies can be explained by differences between the burn protocols utilized. Our protocol involves burning 20% TBSA by immersing the dorsum of the mice in 98 °C water for 10s, whereas the other protocol involves injuring 12.5% TBSA on the dorsum for 10s followed by a 12.5% TBSA on the ventrum for only 2 s, totaling 25% TBSA. Therefore, differences in the severity of burn injury inflicted may result in a delayed induction of Pax7-positive cells. Moreover, the other study showed induction of Pax7 expression that was unable to overcome muscle atrophy. This can be explained by their protocol which involves burning the ventral surface of the mice; this may affect mice mobility and activity post-burn. Another key difference is the technique used to measure Pax7. The other authors examined Pax7 mRNA expression via quantitative polymerase chain reaction (qPCR), whereas we investigated Pax7 localization and protein level via immunohistochemistry and Western blot. The Pax7 mRNA measured by RT-PCR does not necessarily predict its protein level. Finally, there is a difference in the choice of muscle examined; the other study examined the gluteus maximus which has a significantly higher proportion of type IIB fast-twitch fibers than gastrocnemius muscle [54]. Type IIB fibers have been shown to be highly susceptible to muscle wasting, such as in aging [55] and cachexia [56, 57], which may explain the overall atrophic response to burn observed in their study.

Keeping mice unrestrained may have influenced the fast muscle recovery and subsequent regrowth observed in this study. Although a burn affecting 20% TBSA can be life threatening to humans, our group has previously reported that rodents in our protocols 24 h post-burn are highly active and able to resume feeding normally [22]. Moreover, it is plausible that mice become agitated in our burn model leading to more movement in comparison to sham mice. This is noteworthy considering the positive effect of mobilization on muscle growth. Muscle hypertrophy is not typically seen in human burn patients. The quick erosion of lean muscle mass in burn and cancer patients can be further exacerbated by prolonged bed rest in the intensive care unit (ICU). Prolonged immobilization results in loss of body nitrogen and a decrease in skeletal muscle protein synthesis [58, 59]. In contrast, assisting patients to move and exercise during in-hospital care may ameliorate the detrimental effects of bed rest on muscle tissue [60].

## Conclusion

To conclude, we show a transient pattern of muscle atrophy that is tightly associated with an inflammatory cellular and molecular cascade. Our data support the notion that early mobilization of mammalians post-thermal injury can rescue muscle wasting, highlighting the current clinical recommendations. Lastly, targeting NF-κB and/or satellite cells may have therapeutic benefit with regards to muscle atrophy observed after burn injury.

## Additional file

Additional file 1: Immunohistochemistry of F4/80-positive cells in muscle following thermal injury. Representative immunohistochemistry images showing F4/80 staining in sham, 2 days, 7 days, and 14 days post-burn in gastrocnemius muscle. Images were obtained at 20× magnification. (TIF 40911 kb)
