# Supplementary figures and images for: The response of muscle progenitor cells to cutaneous thermal injury
Source: Stem Cell Res Ther. 2017 Oct 17;8:234. doi: 10.1186/s13287-017-0686-z (PMC5646146; doi:10.1186/s13287-017-0686-z)

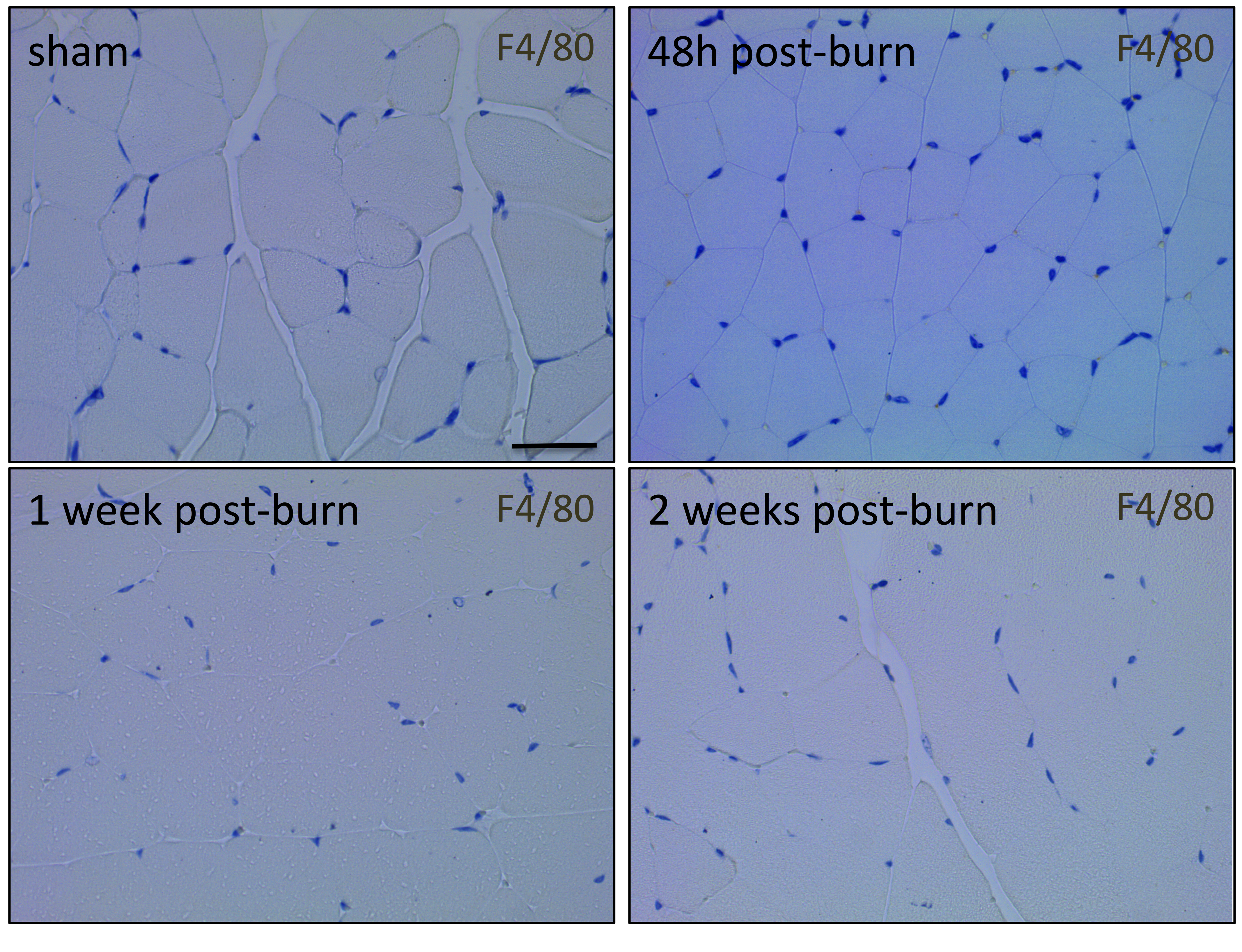

Supplement: Additional file 1: — Immunohistochemistry of F4/80-positive cells in muscle following thermal injury. Representative immunohistochemistry images showing F4/80 staining in sham, 2 days, 7 days, and 14 days post-burn in gastrocnemius muscle. Images were obtained at 20× magnification. (TIF 40911 kb) [file 13287_2017_686_MOESM1_ESM.tif]
